# Monolayer surface chemistry enables 2-colour single molecule localisation microscopy of adhesive ligands and adhesion proteins

**DOI:** 10.1038/s41467-018-05837-7

**Published:** 2018-08-20

**Authors:** Xun Lu, Philip R. Nicovich, Manchen Zhao, Daniel J. Nieves, Mahdie Mollazade, S. R. C. Vivekchand, Katharina Gaus, J. Justin Gooding

**Affiliations:** 1 School of Chemistry, Australian Centre for NanoMedicine and the ARC Centre of Excellence in Convergent Bio-Nano Science and Technology, University of New South Wales, Sydney, NSW 2052 Australia; 2 EMBL Australia Node in Single Molecule Science, School of Medical Sciences and the ARC Centre of Excellence in Advanced Molecular Imaging, University of New South Wales, Sydney, NSW 2052 Australia; 3 Present Address: Allen Institute for Brain Science, Seattle, WA 98109 USA

## Abstract

Nanofabricated and nanopatterned surfaces have revealed the sensitivity of cell adhesion to nanoscale variations in the spacing of adhesive ligands such as the tripeptide arginine-glycine-aspartic acid (RGD). To date, surface characterisation and cell adhesion are often examined in two separate experiments so that the localisation of ligands and adhesion proteins cannot be combined in the same image. Here we developed self-assembled monolayer chemistry for indium tin oxide (ITO) surfaces for single molecule localisation microscopy (SMLM). Cell adhesion and spreading were sensitive to average RGD spacing. At low average RGD spacing, a threshold exists of 0.8 RGD peptides per µm^2^ that tether cells to the substratum but this does not enable formation of focal adhesions. These findings suggest that cells can sense and engage single adhesive ligands but ligand clustering is required for cell spreading. Thus, our data reveal subtle differences in adhesion biology that may be obscured in ensemble measurements.

## Abstract (web summary)

To date, the precise localisation of ligands and adhesion proteins are determined in two parallel characterization setups. Here, the authors report a self-assembled monolayer chemistry for indium tin oxide surfaces allowing single molecule localisation microscopy (SMLM) imaging of ligands and adhesion proteins in a single experiment.

## Introduction

Sophisticated nanofabrication tools such as photolithography and electron beam lithography have allowed researchers to mimic and modulate the chemistry and topography of adhesive ligands found in the extracellular matrix in vivo on substrata for ex vivo studies^1–3^. For example, colloid lithography and block copolymer micelle nanolithography can create repeating patterns on two-dimensional surfaces with the aid of nanoparticles that self-assemble into monolayers or within diblock copolymers^4,5^. Such nanofabricated surfaces have provided novel insights into how cell adhesion^6,7^, differentiation^8–10^, proliferation^11,12^, signalling^13^ and migration^14,15^ are influenced by environmental parameters such as ligand spacing^16^. For example, in pioneering work, Spatz and colleagues showed that the formation of stable focal adhesions requires interligand spacing of RGD-containing peptides of <70 nm and that cell polarisation and migration is sensitive to even nanometre variation in ligand spacing^17–20^. It is currently thought that rather than ligand availability per se, it is the nanoscale clustering of adhesive ligands that is the minimal requirement for cell attachment to the substrate and focal adhesion maturation^21,22^. By pairing nanopatterned surfaces with molecular tension probes, Liu et al. recently demonstrated that sensing of ligand spacing by cells is dependent on the forces generated by the actomyosin cytoskeleton and transmitted to integrin receptors, suggesting that clustering of ligands and adhesion proteins drives stable attachment^23^.

Single molecule localisation microscopy (SMLM) technologies^24^, such as (fluorescent) photoactivated localisation microscopy ((f)PALM)^25,26^, (direct) stochastic optical reconstruction microscopy ((d)STORM)^27,28^, point accumulation for imaging nanoscale topography (PAINT)^29^, and ground-state depletion followed by individual molecule return (GSDIM)^30^, have enabled the precise mapping of protein clusters on the cell surface. This is because SMLM generates images from the molecular coordinates of individual fluorescence events that are temporally segregated during data acquisition^24^. The distribution and clustering can then be quantified with point-pattern algorithms^31^ such as Ripley K-function^32^, pair correlation analysis^33^ and DBSCAN (density-based spatial clustering application with noise)^34^. SMLM imaging and cluster analysis approaches have thus enabled detailed mapping of cluster morphologies and function in a range of cell types^24,35^. An important insight of SMLM is that mature focal adhesions are not homogeneous structures but consists of elongated substructures^36,37^ with single adhesion proteins diffusing in and out of mature adhesions^38,39^. In contrast, nascent adhesions are discreet entities of ~100 nm in diameter containing ~50 activated integrins^40^. These studies highlight the power of SMLM for adhesion biology and the diversity of adhesive studies in adherent cells. To what extent ligand clustering determines the nanoscale architecture of adhesive structures in cells is currently not known.

It would be a logical extension to combine nanofabricated substrates with SMLM imaging and cluster analysis. However, many types of nanofabricated substrates are incompatible with SMLM, as they do not have the optical requirements for single molecule fluorescence^41^. Indeed, in the field of adhesion biology, surface characterisation and cell measurements are often conducted in separate parallel experiments^2^. This means that the average surface parameters such as average ligand spacing are used to interpret the biological responses and inversely, cell behaviours are averaged over large surface areas. This puts additional constraints on the nanofabrication methods in terms of reproducibility between samples and across surface areas, and potentially masked heterogeneity in cellular responses^22^. The separation of surface characterisation and cell experiments has also made it challenging to measure the cellular sensitivity to low ligand densities, or even individual ligands, as this would require the simultaneous detection of the location of the rare ligands and the organisation of cellular proteins on the nanometre scale.

The characterisation of interfaces between substratum and cells has been an enduring challenge in surface science^2,3^. Methods such as X-ray photoelectron spectroscopy (XPS) and reflectometry can give exquisite detail on the coupling yields in forming these interfaces, the density of components in the monolayer and the even the thickness of the layers with sub-nanometre precision^42^. However, as powerful as these methods are, they all provide average information over a larger surface area without providing information of the location of individual ligands. Scanning probe microscopy methods in contrast can provide such localised information^43^ but only prior to the incubation with cells. In the absence of alternative surface characterisation technologies^42^, we were motivated to develop SMLM-compatible surfaces on which ligand spacing could be varied and directly measured after cells were seeded onto the surface.

Here we report a method that gives precise presentation of adhesive ligands to cells, using self-assembled monolayer chemistry on indium tin oxide (ITO) surfaces^44,45^, and is compatible with SMLM imaging of ligands and adhesion proteins. The surface chemistry allowed us to vary average RGD spacing over a wide range. We performed both ensemble measurements such as average number of RGD peptides per area and average number of cells per area, as well as single cell measurements such as the number of RGD beneath individual cells. Further we measured the RGD density in and out of focal adhesions in the same cells. We found that while adhesion of NIH-3T3 cells requires a minimal average density of RGD peptides, once cells adhered and spread, focal adhesions form independently of the position of RGD peptides on the surface, meaning the location of adhesion structures in spread cells does not correlate with variations in local RGD density beneath the cell. This suggests that under these conditions, ligand availability is not limited in the cellular response and formation of mature focal adhesion is likely to be a cell-intrinsic process. In contrast, on surfaces with very low average RGD densities, we found evidence that NIH-3T3 cells can sense and engage individual RGD peptides that allows cells to tether to the substratum but do not spread or form focal adhesions. The described approach is not just a means to simultaneously characterise cell adhesion and ligand distribution with SMLM, but also illustrates that SMLM can be used to characterise biointerfaces at the molecular level^46^. Such biointerfaces are pivotal in sensors, biomaterials and model surfaces for cell biology^42^.

## Results

### Indium tin oxide (ITO) surfaces are suitable for SMLM

**Fig. 1:**
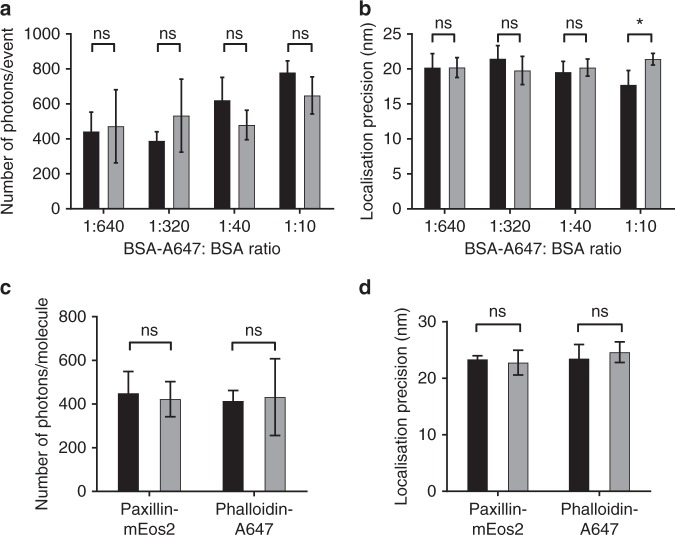
dSTORM data quality is similar on ITO and glass surfaces. **a**, **b** BSA and BSA labelled with Alexa Fluor 647 (BSA-A647) were adsorbed onto ITO (black bars) and glass (grey bars) surfaces at the indicated ratios and imaged with dSTORM under identical conditions. Fluorescent events were identified and grouped as described in the Methods to extract the number of photons (**a**) and the localisation precision (**b**) as a function of labelled BSA. **c**, **d** NIH-3T3-cells-expressing paxillin fused to mEos2 were plated onto ITO (black bars) and glass (grey bars) surfaces and stained with phalloidin conjugated to Alexa Fluor 647. PALM and dSTORM images were recorded under identical imaging conditions for both surfaces and the number of photons per molecule (**c**) and localisation precision (**d**) calculated. Bars and error bars in **a**−**d** indicate average and standard deviation, respectively, of *n* = 5 (**a**, **b**) and *n* = 4 (**c**, **d**) independent experiments; ns not significant; *P ≤ 0.05 (two-tailed *t* test assuming equal variance)

Self-assembled monolayers have been shown to be exceedingly successful in providing molecular level control over ligand presentation in biointerfaces as they are often stable in biological media, have antifouling properties (i.e. prevent nonspecific adsorption) and offer precise control over the number of coupling points to which ligands are attached^2^. To date, the dominant self-assembled monolayer systems are alkanethiols on gold, alkenes and alkynes on silicon and organosilanes on glass^42^. The challenge is that neither gold and silicon are compatible with SMLM while the organosilane systems for modifying glass surfaces are prone to forming multilayers and can be unstable in biological media. This means that unambiguous presentation of ligands on glass is not achieved^42^. We have addressed this conundrum by developing organophosphonate self-assembled monolayer chemistry for ITO surfaces^44,45,47^. However, we first evaluated that the optical properties of ITO surfaces were suitable for SMLM. Alexa Fluor647-labelled bovine serum albumin (BSA-A647) and unlabelled BSA were adsorbed on glass and ITO surfaces, which resulted in the same number of photons emitted from the fluorophores on each surface and gave a localisation precision of ~20 nm on both surfaces for a range of BSA-A647 densities (Fig. 1a, b). Similarly, paxillin fused to the photo-activatable fluorescent protein mEos2 (paxillin-mEos2) expressed in NIH-3T3 cells and phalloidin conjugated to A647 yielded similar photon numbers and localisation precisions in cells on ITO versus glass surfaces (Fig. 1c, d). These results confirmed that ITO surfaces can be used for SMLM.

### Self-assembled monolayer chemistry for ITO surfaces

**Fig. 2:**
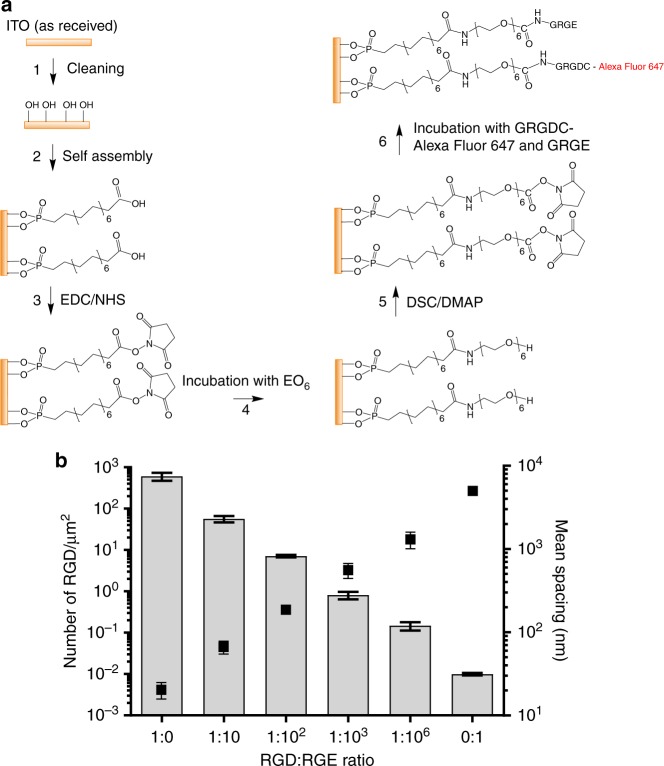
Multistep surface chemistry to functionalise ITO surfaces to control average RGD spacing. **a** Schematic of the formation of the interface where the surface density of RGD peptides attached to the otherwise cell-inert surface (facilitated by hexa(ethylene oxide) molecules, EO_6_) was controlled by controlling the ratio of the molar ratio of GRGDC-Alexa Fluor 647 (RGD) and unlabelled, nonfunctional GRGE (RGE) peptides. **b** RGD peptides imaged by dSTORM showing the number of RGD per μm^2^ on functionalised ITO surfaces. RGD density was determined from SMLM data of *n* = 4 independent experiments. Mean spacing was calculated from RGD densities, assuming a spatially random distribution

We developed the self-assembled monolayer chemistry for ITO where the biointerface is formed in multiple steps as shown in Fig. 2a. The base monolayer was 16-phosphohexadecanoic acid to which hydroxyl-terminated 1-aminohexa(ethylene oxide) was attached. The close packing of the monolayer, and hence compatability with building well-defined interfaces, was verified electrochemically (Supplementary Fig. 1) by showing that redox species in solution could not access the underlying ITO surface and hence redox peaks were absent in the cyclic voltammetry. To this layer, the peptides GRGDC, conjugated to Alexa Fluor 647 (RGD-A647), and nonlabelled RGE-containing peptides were coupled. The cell adhesive ligand density on the surface can be varied by coupling different ratios of adhesive ligands (RGD) to nonfunctional ligands to the surface (RGE). Importantly, although they have different cell adhesive properties, RGD and RGE peptides otherwise have very similar surface properties and hence changing the ratio of the two peptides does not alter the surface other than in terms of cell adhesion^48^. The surfaces were characterised extensively with cyclic voltammetry and XPS from which an average coupling yield of peptides per phosphohexadecanoic acid was estimated to be 12.2% (Supplementary Fig. 2). Since nonfunctional versions are not available for all ligand−receptor pairs, this multistep surface chemistry can be easily adapted to adjust the number of surface bound ligands by diluting the number of hydroxyl-terminated coupling points with methoxyl-terminated hexa(ethylene oxide) molecules, as we have done previously^13^. It should be noted that we observed that fluorescently labelled RGD aggregates on the surface when it was prepared in the absence of the nonfunctional RGE.

Next, we used SMLM to estimate the relative number of RGD-A647 peptides on surfaces with various ratios of RGD-A647 to GRGE peptides (Fig. 2b). This resulted in surfaces with a wide range of RGD densities from 0.01 to 600 molecules/µm^2^, which—assuming a spatially random distribution—correspond to an average RGD-to-RGD spacing of 5  µm to 20.3  nm. We next seeded NIH-3T3 cells onto the functionalised ITO surfaces for 2  h, followed by fixation and fluorescence imaging. As reported previously^13^, the average RGD spacing controls both the average number of cell adhering to these surfaces and cell spreading (Supplementary Fig. 3). It is notable that in ensemble measurements, the surfaces with no RGDs effectively prevented nonspecific adhesion of cells (Supplementary Fig. 3a), while low densities of RGDs, i.e. 1:10^3^ and 1:10^6^ RGD:RGE surfaces, also impaired cell spreading (Supplementary Fig. 3b). Thus, the functionalised ITO surfaces recapitulated the sensitivity of NIH-3T3 cells to RGD spacing that was previously reported for other cell types and surfaces^13,17,18,20^.

### Focal adhesions on RGD-modified ITO surfaces

**Fig. 3:**
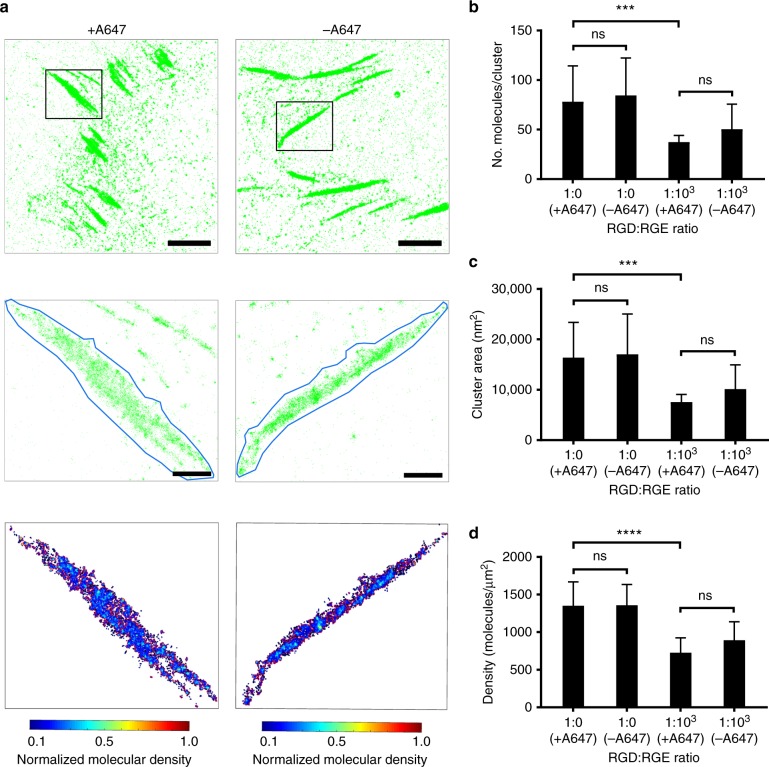
Paxillin-mEos2 clusters on RGD-A647- and RGD-functionalised ITO surfaces. **a** PALM images of paxillin-mEos2 in NIH-3T3 cells adhering onto ITO surfaces that were functionalised with RGD-A647 (left) and unlabelled RGD (right). Top: PALM images of paxillin-mEos2; scale bar = 5 µm. Middle: zoomed regions (black boxes in top row) of an individual adhesive structure with the region for analysis traced (blue line); scale bar = 1 µm. Bottom: paxillin-mEos2 clusters inside identified adhesive structures; colours indicated molecular densities. Images are representative images of *n* = 3−5 independent experiments. **b** Number of paxillin-mEos2 in clusters, **c** cluster area, and **d** density of paxillin-mEos2 molecules inside adhesive structures in NIH-3T3 cells on ITO surfaces with 1:0 and 1:10^3^ of RGD-A647:RGE or RGD:RGE. Paxillin-mEos2 clustering inside adhesive structures was dependent on the RGD density on the surface, but not affected by A647 fluorophore labelled on RGD peptides; ns not significant (*P* > 0.05), ***P < 0.001 and ****P < 0.0001 (two-way ANOVA with Tukey post-testing). Bars and error bars are mean and standard deviation, respectively, *n* = 3−5 independent experiments (including 16−27 independent regions of interest)

Next we imaged adhesive structures of paxillin-mEos2 in NIH-3T3 cells on functionalised ITO surfaces with SMLM (Fig. 3). Since it was previously reported that mature adhesions are not homogenous clusters, but consist of substructures within a mature adhesion^37,39^, we first identified adhesive structures in total internal reflection fluorescence (TIRF) images and then quantified the  properties of paxillin-mEos2 clusters within these adhesive structures after segmentation by DBSCAN^34,35^. This afforded us the opportunity to compare the cluster morphology of paxillin-mEos2 in adhesive structures induced by RGD-A647 peptides compared to RGD peptides without the fluorophore. Given that the number of paxillin-mEos2 in clusters (Fig. 3b), cluster area (Fig. 3c) and the density of paxillin-mEos2 molecules (Fig. 3d) in adhesive structures were similar for RGD-A647- and RGD-functionalised ITO surfaces, we concluded that the A647 fluorophore on RGD peptides did not interfere with adhesion formation. We noticed that the average density of RGD peptides has a significant impact on paxillin clustering with lower average ligand density resulting in fewer paxillin molecules in clusters, smaller clusters and lower paxillin densities in adhesive structures (1:0 RGD:RGE versus 1:10^3^ RGD:RGE surfaces, Fig. 3b−d). These data highlight the details and diversity of adhesive structures SMLM imaging and analysis can reveal.

**Fig. 4:**
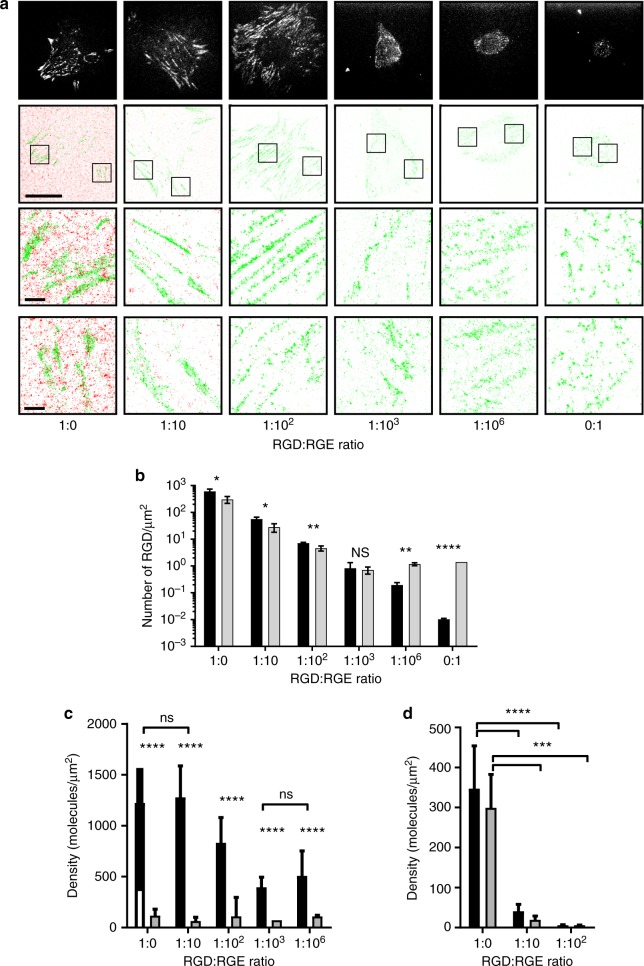
Local RGD density thresholds for adhesion formation and cell tethering to substratum. **a** dSTORM images of RGD ligands on ITO surfaces and paxillin in NIH-3T3 cells. NIH-3T3-cells-expressing paxillin fused to mEos2 were plated onto ITO surfaces that were functionalised with RGD-A647 and RGE peptides at the indicated ratios. Top: TIRF images of paxillin-mEos2; scale bar = 10 µm. Middle and bottom: Merged PALM images of paxillin-mEos2 (green) and dSTORM images of RGD-A647 peptides (red); scale bar = 10 µm. Zoomed regions of individual adhesions are shown in the second and third row; scale bar = 1 µm. Images are representative images of *n* = 3−4 independent experiments. **b** RGD density averaged over the entire surface (black bars) and averaged over the area occupied by cells (grey bars) obtained from dSTORM images of RGD-A647 on ITO surfaces with various ratios of RGD-A647:RGE. Bars and error bars are mean and standard deviation, respectively of *n* = 5 independent experiments; ns not significant; *P ≤ 0.05, **P < 0.01, ***P < 0.001 and ****P < 0.0001 (paired *t* test). **c** Density of paxillin-mEos2 in adhesive structures (black bars) and outside adhesive structures (grey bars) in NIH-3T3 cells on ITO surfaces with various densities of RGD-A647:RGE. Paxillin-mEos2 densities in versus out of adhesive structures were significantly different on all surfaces (****P < 0.0001, unpaired *t* test) while paxillin-mEos2 inside adhesive structures was dependent on the RGD-A647:RGE ratio, except where indicated as ns, not significant (one-way ANOVA with Tukey post-testing). **d** Density of RGD-A647 in adhesive paxillin-mEos2 structures (black bars) and outside adhesive structures (grey bars) in NIH-3T3 cells on ITO surfaces with various densities of RGD-A647:RGE. RGD-A647 densities in versus out of adhesive structures were not statistically significant for all surfaces (unpaired *t* test, not shown) while RGD-A647 densities on 1:0 RGD:RGE surfaces was significantly higher in and out of adhesive structures compared to other presented (***P < 0.001, ****P < 0.0001, one-way ANOVA with Tukey post-testing). In **b**−**d**, data are average and standard deviation from *n* = 3−4 independent experiments

Finally, we plated paxillin-mEos2-expressing cells on RGD-A647-modified surfaces and obtained SMLM images of both the individual RGD peptides and the adhesion protein paxillin-mEos2 with the two-colour SMLM (Fig. 4a). Cells on surfaces with RGD:RGE ratios of 1:0, 1:10 and 1:100 formed visible focal adhesions. On 1:10^3^ and 1:10^6^ RGD:RGE surfaces, a few cells adhered, but did not spread; those cells had many smaller, less pronounced adhesion structures compared to surfaces with higher RGD densities. On the 0:1 RGD:RGE surfaces, onto which only a few RGD-A647 peptides adsorbed nonspecifically, hardly any cells adhered, and those that did were round with no visible adhesive structures (Fig. 4a, Supplementary Fig. 3b).

Although the monolayer chemistry limits nonspecific adsorption, and affords control over average RGD density, individual RGD-A647 peptides were not completely randomly distributed. A Ripley *K*-function analysis revealed spatial heterogeneities that peaked on the 60–160 nm spatial scale (Supplementary Fig. 4). These local heterogeneities in RGD ligands could be caused by irregular protrusions in the underlying ITO coating that exhibit a similar size range^44^. It was noticeable that the nonhomogeneity of the RGD distributions (reflected in the *L*(*r*)-*r* values, Supplementary Fig. 4) was greater at low average RGD densities. This raises the question whether NIH-3T3 cells adhered and formed adhesive structures predominantly at sites where local RGD density was higher, a question that can only be addressed with simultaneous imaging of RGD ligands and adhesion molecules. We thus compared (i) the density of RGD beneath cells versus the surface average and (ii) RGD and paxillin densities inside and outside of adhesive structures.

We first noticed that NIH-3T3 cells only adhered on areas of the ITO surfaces that had >0.8 RGD peptides per µm^2^ irrespective of the average surface density (Fig. 4b). It was particularly noticeable on 1:10^6^ RGD:RGE surfaces and 0:1 RGD:RGE surfaces that those average RGD densities were much lower than 0.8 RGD peptides per µm^2^. Interestingly, the 1:10^3^ RGD:RGE surfaces—surfaces that supported cell adhesion, but limited cell spreading (Supplementary Fig. 3)—had an average RGD density of 0.8 RGD peptides per µm^2^. Thus, it appears that cells had detached from areas with less than 0.8 RGD peptides per µm^2^. However, the nonrandom distributions of RGD peptides meant that same cells could still be tethered to surfaces with lower average ligand spacing. This illustrates that local RGD density rather than average RGD density is the biological relevant parameter for cell interactions with the substratum.

We also examined the density of paxillin molecules (Fig. 4c), the size of paxillin clusters and the number of paxillin molecules in clusters (Supplementary Fig. 5a-b) in and out of adhesive structures by manually identifying adhesive structures based on the TIRF images of paxillin-mEos2, as done above. As expected, paxillin density was vastly higher in adhesive structures as compared with regions outside adhesive structures (Fig. 4c). On surfaces with high average RGD densities (i.e. 1:0, 1:10 and 1:100 RGD:RGE surfaces), the RGD cluster sizes were similar (Supplementary Fig. 5a-b) suggesting that at these average RGD densities, NIH-3T3 cells formed mature focal adhesions. Surprisingly, RGD densities on these surfaces were not significantly different in and out of adhesive structures (Fig. 4d), indicating that cells did not place adhesive structures onto individual RGD peptides. This strongly suggests that local RGD density, but not the positioning of RGD peptides beneath the adherent cells, was the determining factor in focal adhesion formation.

On surfaces with low average RGD densities and on which cell spreading was limited (i.e. 1:10^3^, 1:10^6^ and 0:1 RGD:RGE surfaces), few paxillin clusters formed and clusters were smaller (Supplementary Fig. 5a-b). Here too RGD densities in and out of adhesive structures were similar. A colocalisation analysis^31,49^ revealed that the percentage of paxillin molecules that colocalised with RGD peptides was not significantly different in and out of adhesive structures (Supplementary Fig. 5c), confirming that the position of adhesive structures within cells did not correlate with the position of RGD peptides beneath the cell. This strongly suggests that once cells had adhered and spread on the surface, the formation of adhesive structure was no longer guided by the position of the adhesive ligands on the surface and may have occurred via a cell-intrinsic process such as the clustering of integrin and other adhesion proteins.

## Discussion

In conclusion, we describe a monolayer chemistry to functionalise ITO surfaces that enables the characterisation of ligand distribution and localisation of adhesion protein in a single experiment via the two-colour SMLM imaging. This affords the opportunity to probe both the local cellular environment and the protein distributions within cells in response to engineered surfaces. By examining surfaces with low average RGD densities, our single molecule imaging experiments revealed that NIH-3T3 cells can tether to the substratum in areas of ~0.8 RGD peptides per µm^2^. This suggests that cells can sense and engage individual adhesive ligands. Interestingly, it was recently reported that during the initial phase of the adhesion process, the force per single integrin of ~3 pN is independent of ligand spacing^23^. A few such low-force integrin−ligand interactions may be sufficient to tether cells to the substratum, but not for cell spreading or the formation of nascent and mature focal adhesions. Indeed, we observed that that 4−7 RGD peptides per µm^2^ was needed for cell spreading and adhesion formation. Such higher local density may facilitate integrin clustering, so that the ligand−integrin interactions can withstand forces of ~ 6 pN (ref. ^23^). The generation and maintenance of such high-force ligand−integrin interactions require that ligands are spaced less than 58 nm apart^23^. In our experiments, the location of mature focal adhesion within a NIH-3T3 cell did not correlate with the positioning of the RGD peptides beneath the spread cells suggesting that once cells adhered and spread, a cell-intrinsic process such as actin-generated forces takes over to form mature adhesion structures with a complex molecular suborganisation^37^. SMLM imaging of both protein clustering in adherent cells on model surfaces and the distribution and location of ligands beneath the cells could thus reveal insights into adhesion biology that may have been obscured when surface characterisation and cell adhesion are examined separately.

## Methods

### Chemicals and materials

Indium tin oxide-coated glass (ITO) coverslips (0.17 mm thickness) were purchased from SPI, USA (6480-AB, 15−30 Ω cm, 0.17 mm thickness) and glass coverslips were purchased from ProSciTech, (Sydney, Australia). All chemicals, unless noted otherwise, were of analytical grade and used as received. Aqueous solutions were prepared with Milli-Q water of 18.2 MΩ cm resistivity. Potassium ferricyanide (K_3_Fe(CN)_6_) used as redox active species in electrochemical experiments was obtained from Sigma-Aldrich (Sydney, Australia). *N,N’*-disuccinimidyl carbonate (DSC), dimethylaminopyridine (DMAP), phosphate-buffered saline (PBS), cysteamine, 4-(2-hydroxyethyl)-1-piperazineethanesulfonic acid (HEPES), glucose, glycerol, horseradish peroxidase (HRP), *N*-hydroxysuccinimide (NHS) and 16-phosphonohexadecanoic acid (PHDA) were obtained from Sigma-Aldrich (Sydney, Australia). The antifouling unit 1-aminohexa(ethylene oxide) was obtained from Biomatrik (China). Dichloromethane, tetrahydrofuran (THF), methanol, acetonitrile, ethyl acetate, potassium carbonate, potassium chloride, sodium hydroxide and sodium dihydrogen phosphate were obtained from Ajax (UK). 1-(3-Dimethylaminopropyl)-3-ethylcarbodiimide (EDC) was obtained from Alfa Aesar (UK). Paraformaldehyde (PFA, 16%), BSA and Alexa Fluor 647 C2 maleimide were obtained from Life Technologies (USA). Alexa Fluor 647-labelled GRGDC peptide and unlabelled GRGD peptide were purchased from Cambridge Research Biochemicals (UK). GRGE peptide was purchased from Genscript (USA). All solvents used were analytical grade unless further indicated. Milli-Q water was used for all aqueous solutions, buffer preparations, rinsing and washing steps.

### Synthesis and surface adsorption of BSA-Alexa Fluor 647

A solution of Alexa Fluor 647 C2 maleimide (10 mM, 10 μL) was added to a solution of BSA (2.5 mg/mL, 625 μL) in PBS. The reaction vessel was sealed and placed in the dark at 4 °C. After 24 h the reaction solution was pipetted into Zeba spin desalting column (7K MYCO, Thermo Fisher Scientific) to remove the excess Alexa Fluor 647 C2 maleimide. The degree of labelling of BSA molecules with Alexa Fluor 647 was 90% as determined by UV-Vis spectroscopy. According to the reaction mechanism, each labelled BSA molecule is labelled only by one Alexa Fluor 647 molecule, as the BSA has only one free cysteine on its surface.

A solution of BSA-Alexa Fluor 647 was diluted to 1000 nM in PBS and mixed with different amount of 1000 nM BSA (unlabelled) to make 0.156, 0.313, 0.625, 1.25, 2.5% BSA-Alexa Fluor 647 samples. A few drops of the solution (50 μL) were then pipetted onto clean glass or ITO-coated glass coverslips; the incubation lasted for 20 min in dark condition at room temperature. The incubated substrates were then gently rinsed with PBS (3 × 15 mL) and checked immediately under dSTORM.

### Preparation and modification of ITO and glass substrates

Glass and ITO substrates were first cleaned in an ultrasonicator with dichloromethane and then with methanol for 10 min each, followed by treatment with 0.5 M K_2_CO_3_ in a 3:1 methanol:Milli-Q water mixture for 45 min under sonication. The substrates were then rinsed with copious amounts of Milli-Q water.

ITO surfaces were modified using the procedure outlined previously^44^ and described here. Clean ITO surfaces were immersed in a PHDA solution (1 mM) in THF for 24 h. Then the substrates were then placed in an annealing desiccator under vacuum and annealed at 200 °C for 48 h to promote stable covalent bonding formation. After annealing, the substrates were then rinsed with copious amount of THF to remove possible multilayers and weak bond molecules. Subsequently, the substrates were thoroughly rinsed with Milli-Q water. The PHDA-modified ITO substrates were immediately immersed in a mixed solution of EDC and NHS solution (5 mM) in water for 1 h to activate the carboxyl groups. The EDC/NHS-activated ITO surfaces were rinsed with Milli-Q water (5 × 10 mL) and immediately incubated in the antifouling unit 1-aminohexa(ethylene oxide) solution (200 mM) in dry acetonitrile for 24 h.

ITO coverslips functionalised with an antifouling layer were incubated in a solution of DSC (100 mM) and DMAP (100 mM) in dry acetonitrile in sealed vials (purged with argon) for 20 h. Samples were then removed from solution and immediately rinsed with dry acetonitrile (3 × 10 mL), ethyl acetate (3 × 10 mL), dichloromethane (3 × 10 mL) and dried with nitrogen. A few drops (100 µL) of GRGE and GRGDC-Alexa Fluor 647 or unlabelled GRGD (RGD-A647:RGE 1:0, 1:10, 1:10^2^, 1:10^3^, 1:10^6^, 0:1; RGD:RGE 1:0, 1:10^3^) solution (5 µg/mL in PBS) were placed on the conductive side and left for 15 min. Samples were then rinsed with PBS and stored in PBS in dark at 4 °C before use.

### Surface characterisation

Electrochemical measurements were performed with a BAS-100B electrochemical analyser (Bioanalytical System Inc., Lafayette, IL) and a conventional three-electrode system, comprising an ITO working electrode, a platinum wire as the auxiliary electrode, and an Ag/AgCl 3.0 M NaCl electrode (CH Instrument, USA) as reference. XPS measurements were taken using an ESCALAB 220iXL spectrometer with Al Kα monochromatic source (1486.6 eV), hemispherical analyser, and multichannel detector. Spectra were analysed using AVANTAGE 4.54 software. The fitting of the spectra was performed by a nonlinear least-squares procedure using simple Lorentzian−Gaussian line shapes; prior to peak fitting, a background subtraction was performed using the Shirley method.

### Cell culture and staining

NIH-3T3 were maintained in Dulbecco’s modified Eagle’s medium (DMEM, Gibco) supplemented with 10% (v/v) FBS, 100 U/mL penicillin, and 100 μg/mL streptomycin, and grown at 37 °C in a 5% CO_2_ standard incubator. Cells were at >90% confluency when trypsinised and incubated on the modified ITO substrates. The substrates were put in a six-well plate and 5 × 10^5^ cells were placed in each well and incubated for 2 h at 37 °C in a 5% CO_2_ humidified incubator. Then the medium was removed and the substrates were rinsed with PBS three times. Then 3 mL (for six-well plate) of 4% PFA was placed on each substrate, and the well plate was kept in dark at room temperature for 15 min and then rinsed three times with PBS. 0.1% Triton X100 in PBS was placed on each substrate at room temperature for 5 min and then the substrates were rinsed with PBS three times. For focal adhesion staining, the substrates were incubated in 1% BSA in PBS for 1 h and rinsed with PBS three times. A 1:200 Phalloidin-Alexa 647 solution in PBS was prepared and added on the substrates and incubated for 30 min in dark at room temperature. Then the substrates were rinsed with PBS for three times and imaged under dSTORM.

### Gene transfection

Lipofectamine^TM^ LTX with PLUS^TM^ reagent was purchased from Life Technologies^TM^. NIH-3T3 cells were trypsinised and counted before transfection. Then 5 × 10^5^ cells were plated in a six-well plate and cultured in a standard incubator until 80% confluent. For each well in the six-well plate, a mixture of 0.5 µg of DNA and 0.5 µL of the PLUS^TM^ Reagent in 100 µL of Opti-MEM^®^ reduced serum medium was added. The resulting solution was mixed gently and incubated for 5–15 min at room temperature. For each well of cells, 5 µL of Lipofectamine^TM^ LTX was pipetted into the solution, mixed gently and incubated for 25 min at room temperature to form DNA-Lipofectamine^TM^ LTX complexes. Then the growth medium was removed from cells and replaced with 3 mL of complete growth medium. One hundred microlitres of the DNA- Lipofectamine^TM^ LTX complexes was added directly to each well and mixed gently by rocking the plate back and forth. Finally, the cells were incubated at 37 °C in a CO_2_ incubator for 24 h post-transfection before assaying for transgene expression.

### Preparation of oxygen scavenging buffer for dSTORM imaging

Oxygen scavenging buffer was prepared in the following way: (1) A base buffer stock containing PBS (1×), HEPES (25 mM), glucose (25 mM) and glycerol (5%) was adjusted to pH 8.0 and filtered through a 0.22 µm filter (Millipore, 47 mm regenerated cellulose); (2) frozen stock solutions of glucose oxidase (GOx, 10 mg/mL in 50 mM phosphate buffer pH 5.1) and HRP (10 mg/mL in 100 mM phosphate buffer pH 6.0) were added to the base buffer solution for a final concentration of 0.05 mg/mL GOx and 0.025 mg/mL HRP; (3) cysteamine hydrochloride solution (1 M) was prepared just prior to the experiment and added to the base buffer containing GO and HRP for a final cysteamine concentration of 10 mM.

### SMLM imaging (dSTORM/PALM)

SMLM was performed on a LSM 7 EYLRA system equipped with 405, 488, 561 and 642 nm lasers and a Plan-Apochromat ×100/1.46 Oil DIC M27 objective lens. Laser power was adjusted and a TIRF angle between 64° and 67° was used for acquisition. Pretreated glass and ITO coverslips were loaded into an 18 mm × 18 mm square Chamlide microscope chamber (Live Cell Instrument, CM-B-30), and were submerged in 500 μL of oxygen scavenging buffer. In a typical dSTORM experiment, the illumination was focused on an area of ~ 25 μm × 25 μm under continual radiation of 488 and 647 nm laser. 10,000−100,000 frames (or until blinking events ceased) were captured with an exposure time of 30 ms and a camera gain of 100. For a typical PALM experiment, the surface was under continual radiation of 405 and 561 nm laser. A total of 20,000 frames were captured with an exposure time of 30 ms and a camera EM gain of 100.

For imaging cell adhesions and RGD modified on ITO surface, the two-colour imaging (dSTORM/PALM) was applied on the same imaging area. Here, 500 μL of PBS were first loaded into the Chamlide microscope chamber with 15 μL of colloidal spherical gold nanoparticles (From BBI solutions, 250 nm diameter, ~5000 nanoparticles) solution and the location of the transfected cells was identified under 488 nm. Subsequently, this solution was replaced with 500 μL of dSTORM buffer and a dSTORM image of Alexa Fluor 647 was taken under 488 and 647 nm lasers. After dSTORM imaging, a PALM image of mEos2 was then taken under 405 and 561 nm lasers at the same area.

The captured data were analysed using PALM processing algorithms (Zeiss ZEN 2012) as described previously^35,50^ as well as here. After Gaussian filtering, fluorescent blinking events were identified as *I* − *M* > 6S, where *I* is the event intensity, *M* the mean image intensity and *S* the standard deviation of the image intensity. The peak mark size was set at nine pixels for Alexa Fluor 647 and six pixels for mEos2, which allowed for the detection of dye molecules on the surface and minimised counts arising from background noises. Each event corresponding to a point-spread function was fitted to a two-dimensional Gaussian distribution to calculate its centre, accounting for the possibility of overlapping peaks. The localisation precision was calculated based on the definition of Mortensen et al.^51^. Events within a radius of 100 nm were grouped together if they appeared in the same area, last for no more than five frames (on-time) and the gap between blinking events was no more than 50 frames (off-gap). To correct for potential sample drift, we used a built-in drift correction function from the Zen software that uses a method based on the triangulation of localisations and a piecewise-linear drift model. Finally, tables containing the *x−y* particle coordinates of each spot detected in the acquisition were generated and used subsequently for DBSCAN and Ripley *K*-function analysis.

### Image analysis

SMLM data were analysed using custom software written in MATLAB (MathWorks).

Ripley *K*-function^32^ was used to determine the extent of clustering of a population of molecules compared to a random distribution at the same density. In brief, the Ripley *K*-function calculates the number of neighbour molecules for each molecule within a given radius *r* corrected by the total density and then the average is calculated for each radius and all molecules. It therefore provides ensemble information on the whole region of interest.

A density-based spatial clustering application with noise analysis (DBSCAN) was used for cluster identification and segmentation^34^. DBSCAN identifies clusters in large datasets of points in a propagative fashion^52^ based on the search radius (*r* = 20 nm) and the minimum number of neighbours (*ε* = 3). The DBSCAN routine is implemented in MATLAB and subsequently coded in C++ and compiled in an MEX file (Matlab executable file) to improve the speed of processing as we are working with large data files.

Two-colour SMLM data were also analysed with a degree-of-colocalisation (DoC) analysis. As previously described^31,35,49^, the local density of each channel and each molecule is calculated at increasing radius size (10–500 nm), providing the density gradient around that molecule for each channel. The two density gradients are tested for correlation with the Spearman criteria, which score monotonic dependence and corrected with nearest neighbour distance to account for long distance interactions. As a result, each molecule is assigned a DoC value ranging from −1 to 1, with −1 characterising anticolocalisation (or segregation), 0 corresponding to single species and 1 defining high colocalisation. A paxillin molecule was regarded as colocalised with an RGD peptide if its DoC value was ≥0.4.

The GUI enabled drawing of multiple subregions within each image and returned the output of the DBSCAN and DoC analysis for each region individually. Cell outlines and regions representing adhesive structures were manually segmented using TIRF images. Regions representing nonadhesive structures were inside in the cell outline and not belonging to the adhesion structures.

### Statistical analysis

All statistical analyses were performed with Prism software (Graphpad Software). Unless stated otherwise, all data are presented as mean ± standard deviation from at least three independent experiments. Unpaired two-tailed Student’s *t* tests were performed for testing of statistical significance between two populations. Multiple comparisons were made by either one-way or two-way analysis of variance (ANOVA) followed by Tukey’s test. In statistical analysis, *P* > 0.05 is indicated as not significant (ns), whereas statistically significant values are indicated by asterisks as follows: *P ≤ 0.05, **P < 0.01, ***P < 0.001 and ****P < 0.0001.

### Code availability

Custom software and computer codes used in this study are available from the corresponding authors upon request.

### Data availability

The data that support the findings of this study are available from the corresponding authors upon reasonable request.

## Electronic supplementary material

Supplementary Information
