# Supplementary material for: Monolayer surface chemistry enables 2-colour single molecule localisation microscopy of adhesive ligands and adhesion proteins
Source: Nat Commun. 2018 Aug 20;9:3320. doi: 10.1038/s41467-018-05837-7 (PMC6102261; doi:10.1038/s41467-018-05837-7)
Supplement: Supplementary file 1 — Supplementary Information [file 41467_2018_5837_MOESM1_ESM.docx]

Supplementary Information

**Monolayer surface chemistry enables single molecule localisation microscopy (SMLM) imaging of ligands**

Xun Lu^a^, Philip R. Nicovich^b,c^, Manchen Zhao^a^, Daniel J. Nieves^b^, Mahdie Mollazade^b^, S.R.C. Vivekchand^a^, Katharina Gaus^b,*^, J. Justin Gooding^a,*^

^a^School of Chemistry, Australian Centre for NanoMedicine and the ARC Centre of Excellence in Convergent Bio-Nano Science and Technology, University of New South Wales, Sydney, 2052, Australia

^b^EMBL Australia Node in Single Molecule Science, School of Medical Sciences and the ARC Centre of Excellence in Advanced Molecular Imaging, University of New South Wales, Sydney, 2052, Australia

^c^Present address: Allen Institute for Brain Science, Seattle, Washington, 98109, USA


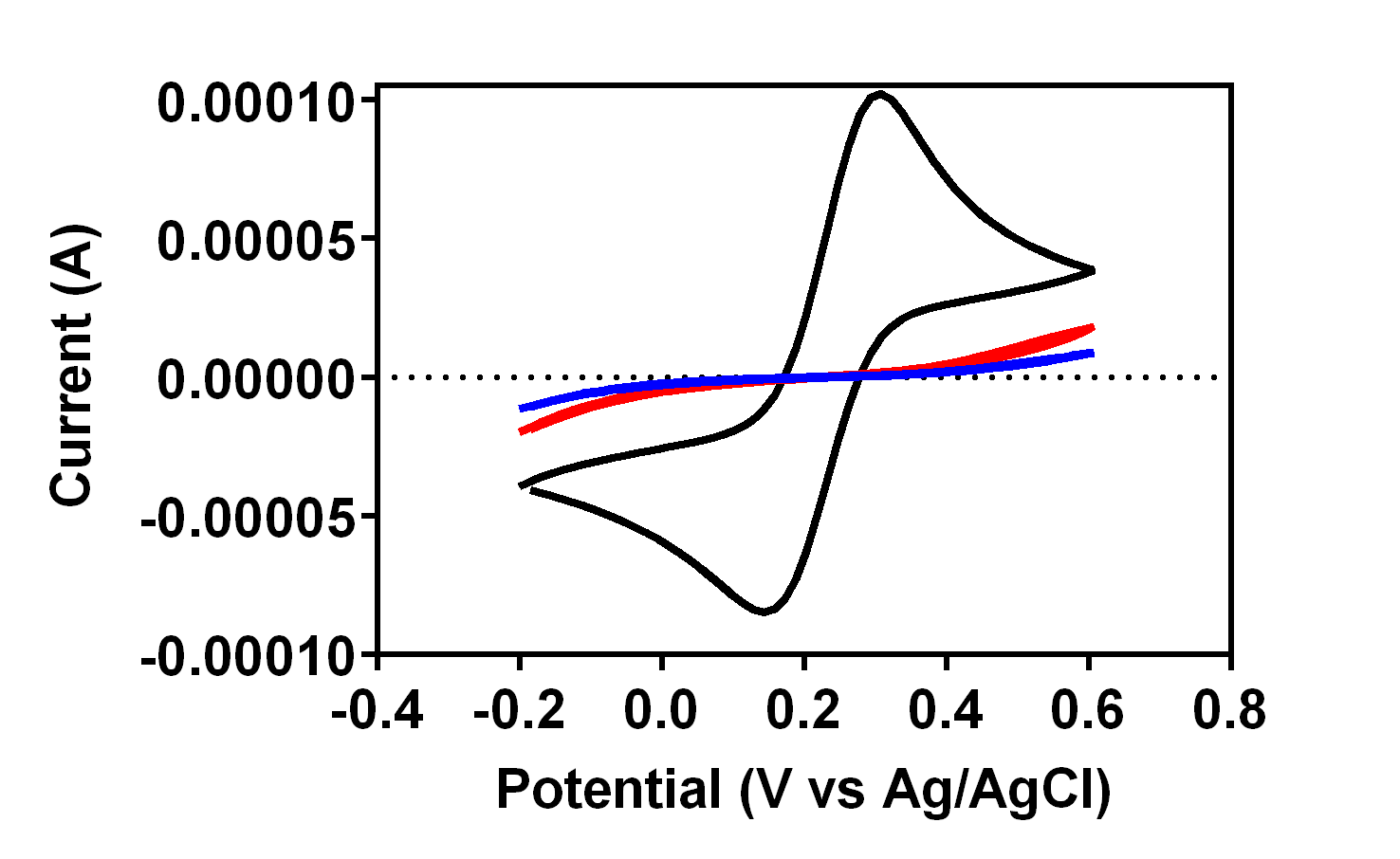


Supplementary Figure 1. Cyclic voltammogram analysis of modified ITO surfaces. Cyclic voltammograms were recorded in 0.1 M KCl aqueous solution containing 1 mM K_3_[Fe(CN)_6_] and 1 mM K_4_[Fe(CN)_6_] at the scan rate of 100 mV/s with an unmodified ITO (black line), an ITO surface after 1-aminotetra(ethylene oxide) attachment (blue line) and an ITO surface after final GRGE and GRGDC-Alexa Fluor 647 attachment (red line), showing the monolayer chemistry prevented solution species reaching the electrode surface (passivated) and the passivation remained with further modification of the monolayer on the ITO surface with peptides.


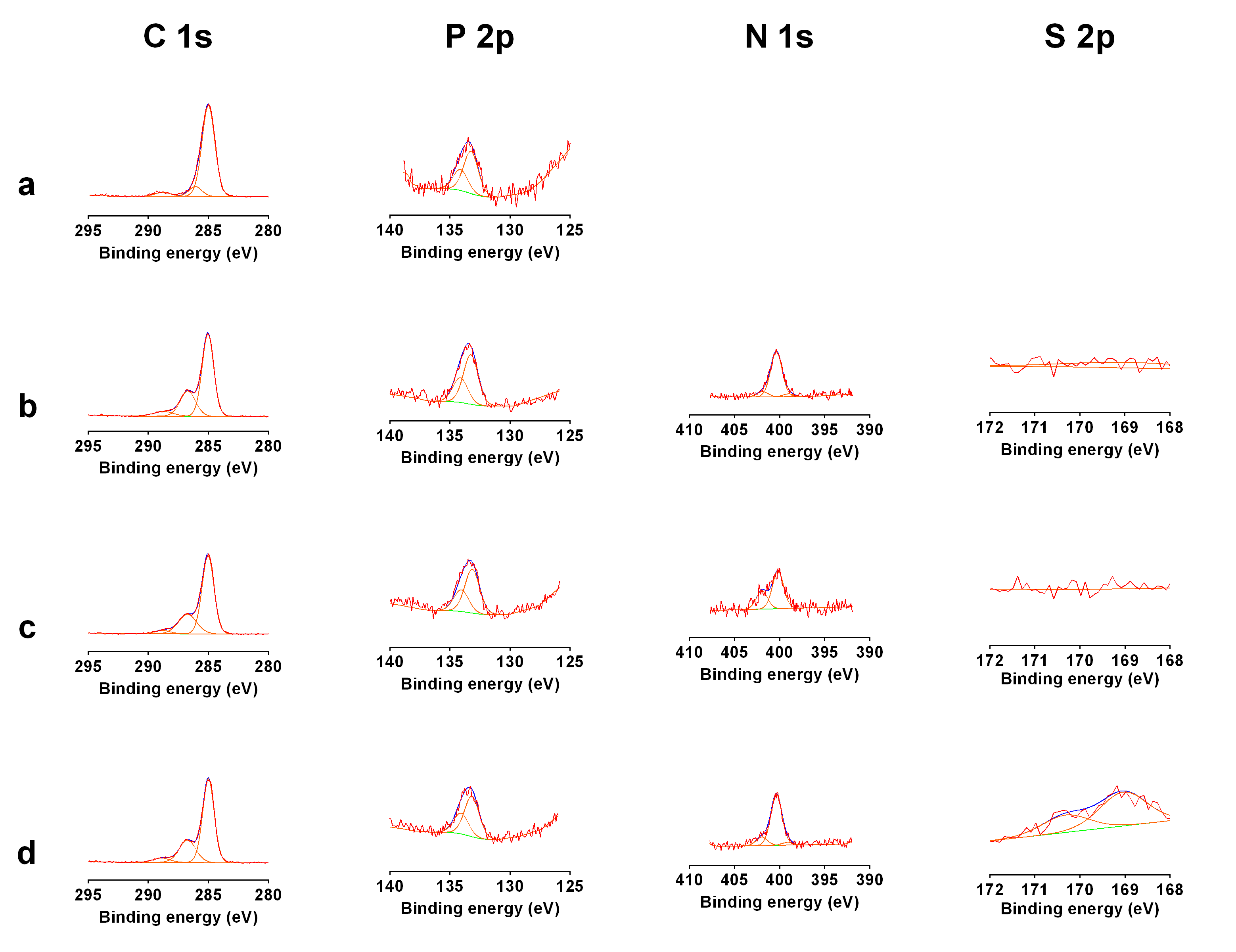


**Supplementary Figure 2. XPS characterization of the surface modification steps.** (**a-d**) C *1s*, N *1s*, P *2p* and S *2p* XPS scans after carboxyl-terminated PHDA self-assembly monolayer modification, step 2 (**a**), after 1-aminohexaethylene glycol attachment, step 4 (**b**), succinimide ester-activated surface after the DSC/DMAP activation reaction, step 5 (**c**), and after GRGDC-Alexa Fluor 647 attachment, step 6 (**d**).

The coupling yield was estimated as follows: The C *1s* scan in (**a**) was deconvoluted with fitting to three functions: 1) a peak at 285 eV attributed to aliphatic carbon-bonded carbons (C-C), 2) a weak peak at 287 eV corresponding to oxygen-bonded carbon (C-O) originating from the ITO surface and 3) another weak signal at 289.5 eV assigned to the carbon atom within the carboxylic group (O-C=O). The P *2p* narrow scan showed a peak which was assigned to the phosphorus in the phosphonate, indicating the formation of PHDA on ITO surface. However, the N *1s* and S *2p* narrow scans suggested there was no nitrogen or sulphur presented in PHDA-modified surfaces. Then after attachment of the 1-aminohexa(ethylene oxide) in step 4 (**b**), a peak in N *1s* region suggested the presence of nitrogen species on the surface while there was still no S *2p* peak. Moreover, the increase of the C-O peak at 287 eV provides further evidence for the C-O rich ethylene oxide molecule. The coupling yield of the 1-aminohexa(ethylene oxide) to the PHDA-modified surface as per step 4 was determined by comparing the area under the amide peak in the N *1s* region with the phosphonate peak in the P *2p* region. The coupling efficiency for the modification of acid groups with ethylene oxide molecules was 56.14% which is consistent with previous studies where oligo(ethylene oxide) species were coupled to monolayer modified surfaces. Next the coupling yield of attaching the mixture of RGD and peptide to the 1-aminohexa(ethylene oxide) terminated surface was compared by comparing the area of the sulphonate peak, in the S *2p* region, arises from the Alexa Fluor 647 and the amide peaks in the N *1s* region that arise from the amides bonds on the peptides. For the conjugation of the Alexa Fluor 647 labelled GRGDC on the NHS- terminal was 24.43%. Hence the overall coupling efficiency of PHDA molecules are modified with the GRGDC peptide was calculated to be 12.22%. The assumption is made that the coupling of GRGE peptide was identical to the GRGDC.


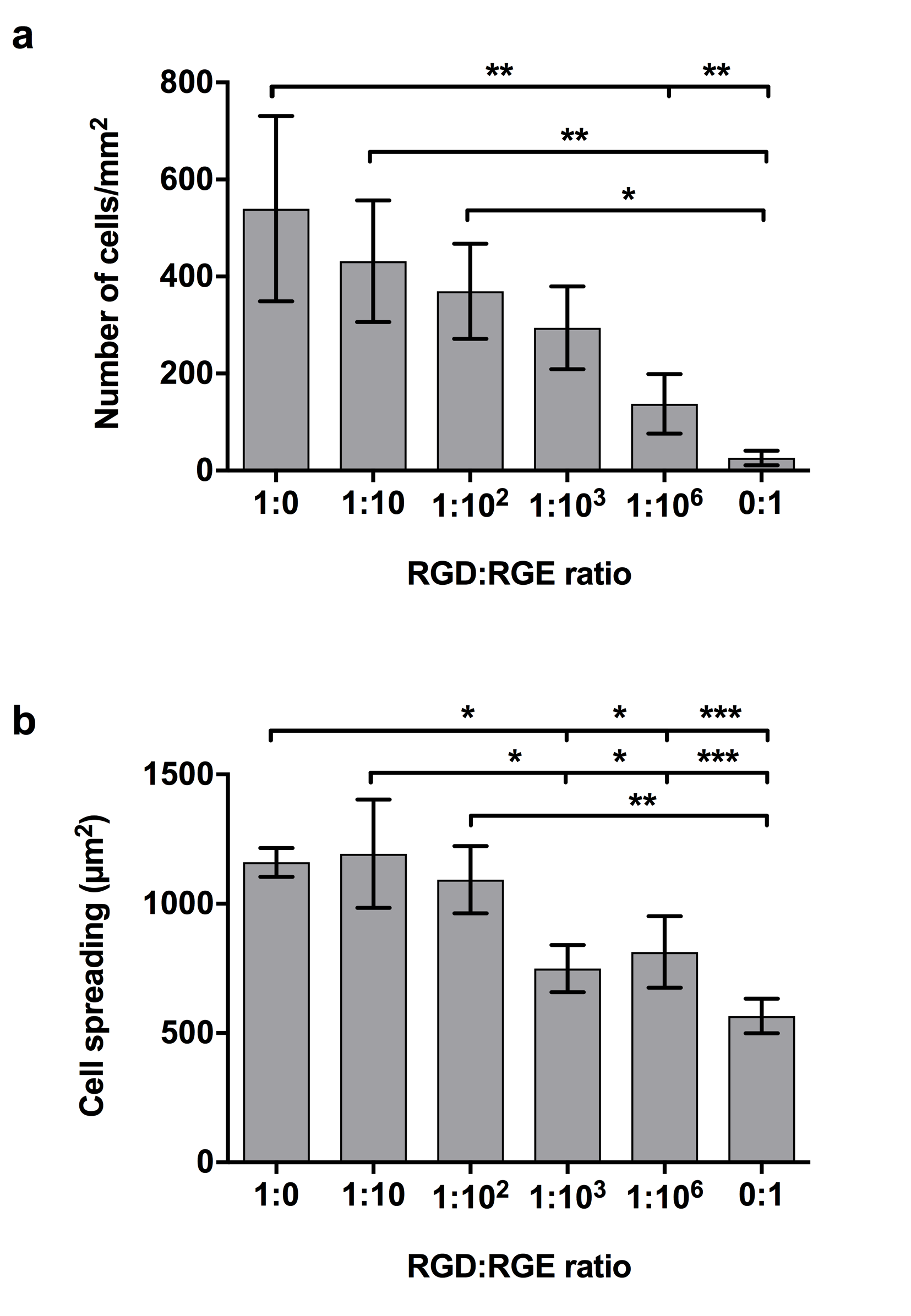


**Supplementary Figure 3. Cell number and average cell area on different densities of RGD-modified ITO surfaces.** Number of cells per mm^2^ and average cell area on ITO surfaces functionalized with various ratios of GRGDC-Alexa Fluor 647 (RGD) and unlabelled GRGE (RGE) peptides. NIH 3T3 cells were plated onto these surfaces for 2 h, fixed, and labelled with phalloidin (F-actin). Cell numbers (**a**) and cell area (**b**) were determined from epifluorescence images using ImageJ. Data are average and standard deviation, respectively, from n = 3-4 independent experiments; ns, not significant (P > 0.05), *P ≤ 0.05, **P < 0.01, ***P < 0.001 and ****P < 0.0001 (two-way ANOVA with Tukey post-testing).


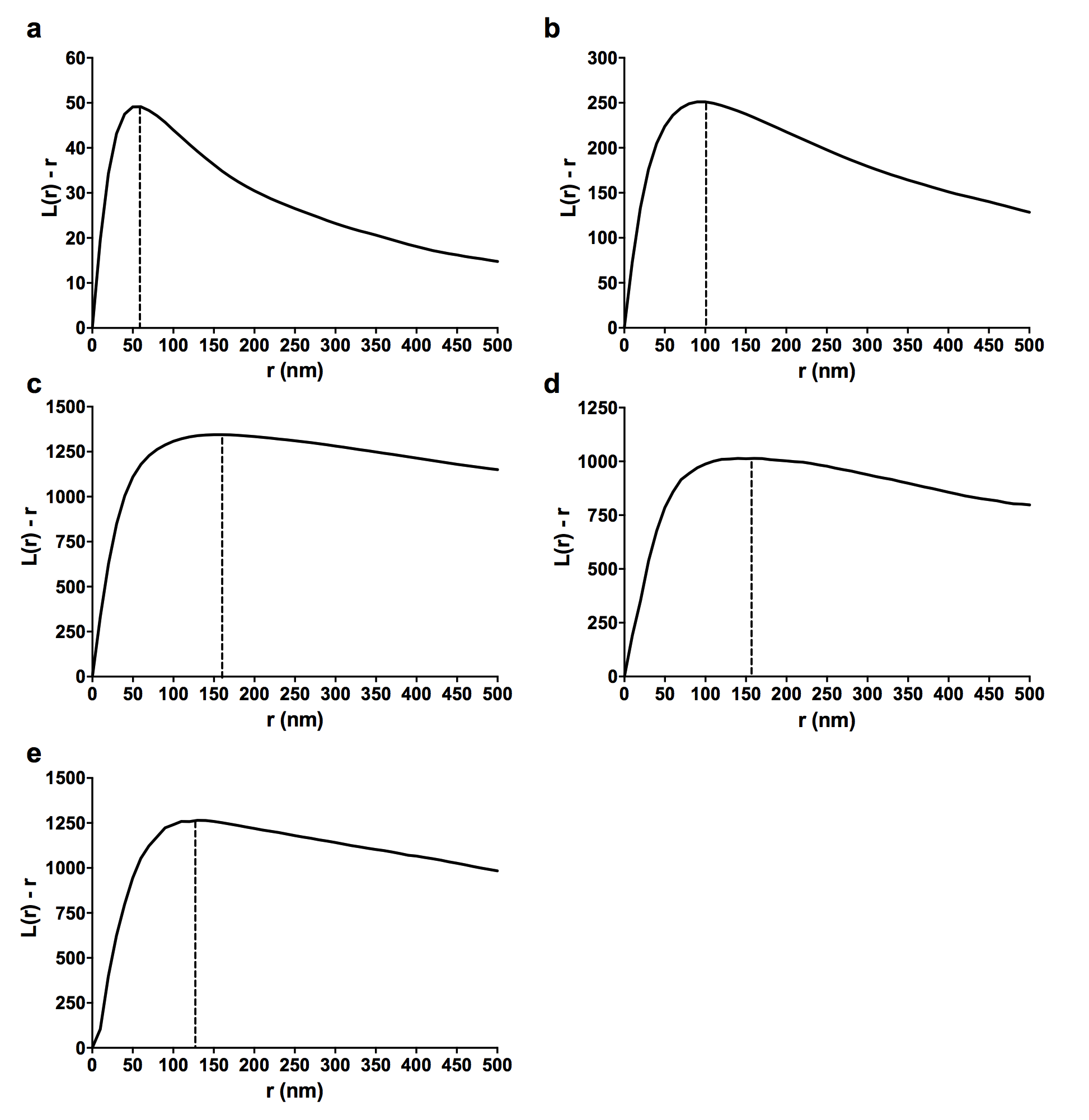


**Supplementary Figure 4. Ripley’s K-function analysis of RGD-modified ITO surfaces.** ITO surfaces were functionalized with self-assembled monolayers as outlined in Supplementary Figure 1 with 1:0 (**a**), 1:10 (**b**), 1:100 (**c**), and 1:10^6^ (**d**) ratios of GRGDC-Alexa Fluor 647 (RGD) and unlabelled GRGE (RGE) peptides RGD peptides imaged by dSTORM. The point distributions of RGD peptides was analysed with Ripley K-function where L(r)-r reports the degree of clustering relative to a random distribution, and r is the radical scale. The Ripley K-function curves are averages of n = 4 independent experiments and peaked (indicated by dashed line) at 60 nm (**a**), 100nm (**b**), 160 nm (**c**), 160 nm (**d**), and 130 nm (**e**).

**
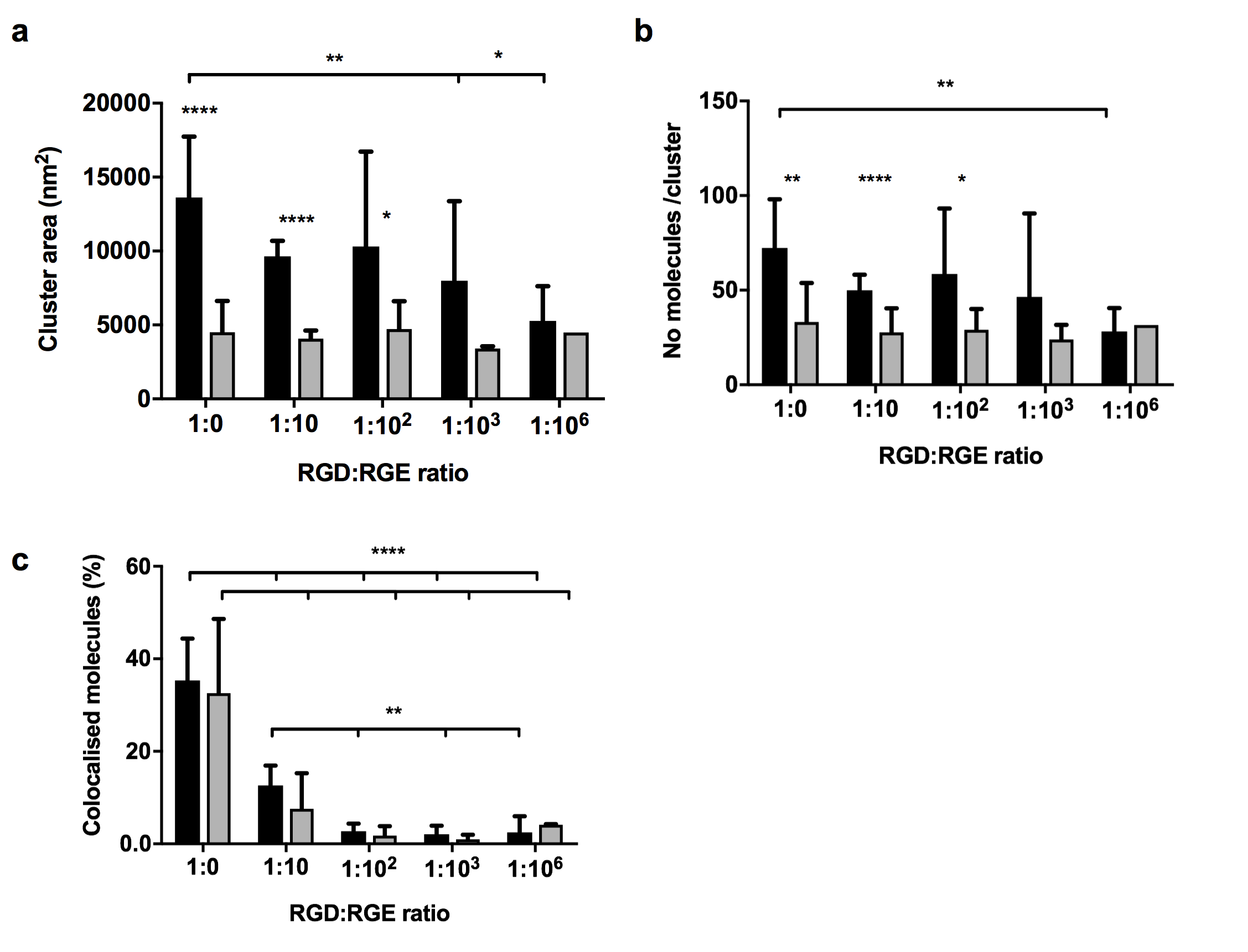
**

**Supplementary Figure 5. Paxillin clustering and co-localisation with RGD peptides.** Cluster analysis (**a-b**) and degree of co-localisation analysis (**c**) of paxillin-tdEOS inside (black bars) and outside (grey bars) of adhesive structure on ITO surfaces with various ratios of ratios of GRGDC-Alexa Fluor 647 (RGD) and unlabelled GRGE (RGE) peptides RGD peptides. 2-colour dSTORM images of paxillin-tdEOS in NIH 3T3 cells and GRGDC-Alexa Fluor 647 (RGD) were analysed with a cluster analysis to extract the cluster area (**a**) and number of paxillin-tdEOS molecules per cluster (**b**), and a degree-of-colocalisation analysis (**c**) as described in *Methods*. Data are average and standard deviation, respectively, from n = 3-4 independent experiments. Comparisons inside and outside of adhesive structures were performed with unpaired t-tests (*P ≤ 0.05, **P < 0.01, ****P < 0.0001 in a-b) and comparisons between surfaces with two-way ANOVA with Tukey post-testing (horizontal bars, *P ≤ 0.05, **P < 0.01, ****P < 0.0001).
